# The Dual Faces of S1P: Orchestrating Immune Responses in Health and Disease

**DOI:** 10.3390/cells15080663

**Published:** 2026-04-09

**Authors:** Stephanie A. Mills, David Barr, Shikhar Mehrotra, Paramita Chakraborty

**Affiliations:** Department of Pharmacology & Immunology, Hollings Cancer Center, Medical University of South Carolina, Charleston, SC 29425, USA

**Keywords:** sphingosine 1-phosphate, S1P receptor signaling, tumor microenvironment, cancer immunology, immunotherapy

## Abstract

Sphingosine 1-phosphate (S1P) is a potent bioactive sphingolipid that plays essential roles in regulating various immune responses, including lymphocyte trafficking, immune cell differentiation, and immunosurveillance. Different immune responses to S1P arise from the diverse Sphingosine 1-phosphate receptors (S1PRs) expressed on the cell surface, shaping unique, context-dependent responses to S1P. Beyond surface receptor engagement, intracellular S1P signaling is also being recognized as a crucial modulator of immune cell responses. Furthermore, the multifaceted S1P signaling axis has emerged as a key regulator of immune responses within the tumor microenvironment (TME), influencing both innate and adaptive immune cell behavior to facilitate tumor progression. A deeper mechanistic understanding of S1P signaling and its impact on immune cell fate is essential for developing novel therapeutic strategies to enhance anti-tumor responses. This review summarizes our current knowledge of how S1P influences immune cell function, with a specific focus on S1PR-dependent and S1PR-independent cellular signaling pathways. We also examine the alterations in immune cell responses that occur within the TME and current therapeutic strategies targeting S1P signaling.

## 1. Introduction

Sphingosine 1-phosphate (S1P), derived from sphingosine, was recognized as a bioactive signaling molecule in the early 1990s [1]. The significance of S1P signaling on human health first became evident with the discovery of the novel immunosuppressive drug FTY720 (Fingolimod, Gilenya, Novartis), a S1P receptor functional antagonist, which triggers lymphopenia and has been used to suppress autoimmune diseases such as multiple sclerosis [2,3,4]. Since then, studies have explored the impact of S1P on both the innate and adaptive immune systems, revealing complex sphingolipid signaling pathways that modulate immune responses. S1P has been recognized as a central regulator of immune cell behavior linking lipid metabolism with immune signaling pathways that control cell trafficking, differentiation, and survival. While early studies focused primarily on S1P gradients regulating lymphocyte trafficking, recent work demonstrates that S1P signaling also modulates immune cell metabolism, transcriptional programs, and functional differentiation within the tumor microenvironment (TME). Depending on receptor expression, cellular source of S1P, and metabolic context, S1P signaling can promote either immune activation or immunosuppression. Understanding this context-dependent signaling network is therefore critical for developing therapeutic strategies targeting the S1P axis in cancer. We therefore focused on advances in the last ten years in our understanding of S1P in the context of immune cell modulation and immunotherapeutic approaches to cancer treatment. We further discuss outstanding questions and gaps in the field regarding S1P and immune modulation, and S1P therapeutic approaches.

## 2. S1P Regulation and Immune Cell Function

### 2.1. S1P Synthesis

In the cell membrane, complex sphingolipids are catabolized to form ceramide, which can then be further deacetylated to form sphingosine, the precursor of S1P [5]. Growth factors and cytokines can then activate sphingosine kinases (SphKs) to mediate the phosphorylation of sphingosine to S1P [6,7,8]. There are two isoforms of SphK in immune cells, sphingosine kinase 1 (SphK1) and sphingosine kinase 2 (SphK2) [9,10,11]. Despite having comparable amino acid sequences, these two isoforms have distinct developmental expression kinetics, subcellular localizations, and different biological purposes [12,13,14,15,16]. SphK1 is localized predominantly in the cytosol, while SphK2 shuttles between the nucleus and the cytoplasm and can be found in the endoplasmic reticulum, mitochondria, and plasma membrane under certain conditions [8,17,18,19]. Consequently, S1P generated by SphK1 is primarily secreted from the cell (reviewed below in S1P secretion) to act in a paracrine and autocrine manner. In contrast, S1P generated by SphK2 in mitochondria and nuclei plays crucial roles in regulating respiration, histone acetylation, gene expression, and telomerase stability in these organelles [20,21,22,23]. Loss of both SphK1 and SphK2 isoforms is embryonically lethal; however, deletion of either enzyme alone does not lead to an aberrantly lethal phenotype, indicating that SphK1 and SphK2 have both distinct and redundant roles in the cell to generate S1P [24,25].

### 2.2. Irreversible and Reversible Breakdown of S1P

Similar to other bioactive mediators, S1P levels are tightly regulated by the balance between its synthesis and breakdown [26,27,28]. S1P phosphatase-1 (SGPP1) and S1P phosphatase-2 (SGPP2) are S1P-specific phosphatases located on the endoplasmic reticulum and are responsible for dephosphorylation of S1P back to sphingosine. Broad lipid phosphate phosphatases (PLPP), meanwhile, are located on the plasma membrane and have broad specificity for multiple lipids, including S1P. PLPPs are responsible for up taking and reversibly recycling extracellular S1P back to sphingosine [29,30,31]. These two enzyme classes can dynamically alter the equilibrium of sphingosine to S1P levels to modulate cell fate [31,32,33]. S1P lyase, also an endoplasmic reticulum-resident enzyme, irreversibly cleaves the C2-3 bond of S1P to generate hexadecenal and phosphoethanolamine [33,34,35]. This reaction transfers hexadecenal from the sphingolipid pathway to the glycerolipid pathway for further conversion to hexadecanoate [36]. Phosphoethanolamine is used for downstream phosphatidylethanolamine synthesis [37,38]. This provides two distinct pathways within the cell for modulating intracellular S1P levels.

### 2.3. S1P Secretion

**Figure 1:**
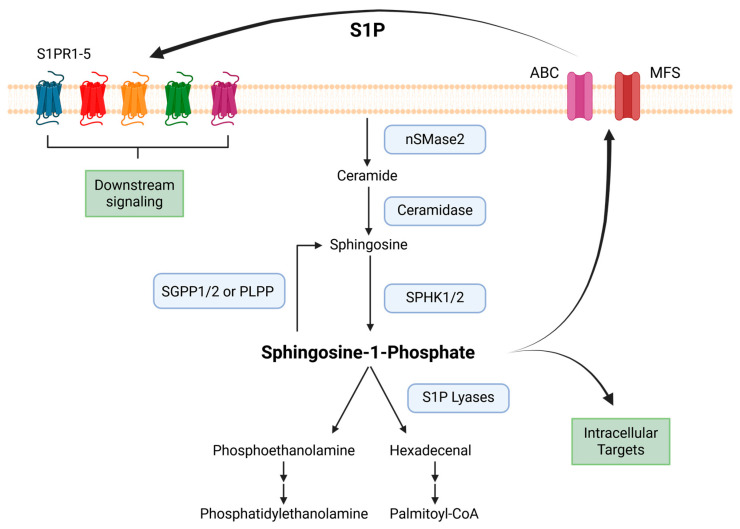
S1P synthesis, degradation, and secretion. Sphingosine 1-phosphate (S1P) can be synthesized through the deacetylation of ceramide to form sphingosine and then the subsequent phosphorylation of sphingosine by SphK1/2. S1P phosphatases (SGPP1/2) and lipid phosphate phosphatases (PLPP) can reversibly dephosphorylate S1P back to sphingosine and are critical modulators of the S1P-to-sphingosine ratio in the cell. S1P lyases can degrade S1P to form intermediate byproducts, phosphoethanolamine and hexadecenal, which can then be shuttled towards different metabolic pathways in the cell. S1P is a critical signaling molecule that can bind to intracellular targets or be secreted extracellularly by ATP-binding cassette (ABC) and Major Facilitator Superfamily (MFS) transporters to activate S1P receptors in an autocrine or paracrine manner, leading to downstream G protein-coupled signaling. Created in BioRender. MEHROTRA, S. (2026) https://BioRender.com/2igvpvs (accessed on 20 January 2026).

Upon synthesis, S1P can function by (1) binding to an intracellular target or (2) exiting and then activating S1P receptors in a paracrine and/or autocrine manner through an inside-out mechanism [34]. S1P cannot spontaneously traverse membranes due to its amphipathic property and therefore must be secreted through active transport. Several families of transporters have been identified, including ATP-binding cassette (ABC) transporters and Major Facilitator Superfamily (MFS) transporters, to mediate the secretion of S1P (Figure 1). These transporters play critical roles in regulating global plasma S1P levels, establishing S1P gradients between tissue compartments, and mediating S1P secretion by different immune populations.

#### 2.3.1. ABC Transporters

ABC transporters were first identified in 2006 to secrete S1P from platelets upon thrombin treatment [35]. Since then, secretion of S1P through ABC transporters has been identified in multiple cell types, including mast cells, astrocytes, and erythrocytes [36,37,38]. These transporters depend on environmental cues, such as ATP and calcium, and are also sensitive to drugs such as glyburide and estradiol [35,39,40]. P-glycoprotein and multidrug resistance protein 1 (MRP1), two ABC transporters, are both expressed by dendritic cells (DC). According to functional descriptions, both transporters are necessary for effective DC and T cell migration. According to Scheper et al., MRP1 is also essential for DC differentiation. Antagonists of the MRP1 transporter inhibit early DC development in vitro. This indicates that specifically, S1P transportation is fundamental in DCs to fulfill essential physiological functions [41]. Mast cells depend on MRP1-mediated export of S1P for chemotaxis toward antigen [38]. Loss of ABC transporters MRP1 or P-glycoprotein, however, does not alter global levels of S1P in murine plasma [37,42,43,44], suggesting that these transporters play more specialized roles in S1P export and autocrine/paracrine signaling. Collectively, these findings indicate that ABC transporters primarily regulate localized S1P gradients rather than systemic S1P levels.

#### 2.3.2. MFS Transporters

Spinster homolog 2 (Spns2) was the first member of the MFS transporters identified to secrete S1P. Spns2 is unique and lacks a typical ATP-binding motif [45,46,47,48]. Osborne et al. first appreciated the importance of Spns2 in zebrafish models on myocardial embryonic development and migration. They found a recessive mutation ‘two of hearts’, later recognized as *Spns2*, led to abnormal heart-tube formation and Cardiac Bifida. Supplementation with exogenous S1P rescued myocardial embryonic development independent of ‘two of hearts’ mutant expression, suggesting Spns2 may be a transporter of S1P [49]. This was later confirmed by endogenously over-expressing zebrafish Spns2-EGFP and tracking the export of tritium-labeled S1P [48]. These studies established Spns2 as a key mediator of S1P export during development. S1P export through Spns2 is an essential metabolic mediator of macrophages. When macrophages lack Spns2, intracellular lactate production increases, thereby enhancing glycolysis. This enhances the proinflammatory response by increasing reactive oxygen species production and has been associated with lethal hyperinflammation in the early stages of sepsis. Decreasing Spns2 compromises the long-term antibacterial capacity of macrophages, leading to severe innate immunosuppression during late-stage infection and highlighting the critical role of Spns2-dependent S1P export in balancing macrophage metabolic and inflammatory responses [50]. Major facilitator superfamily domain-containing protein 2b (MFsd2b) is another MFS protein that exports S1P from platelets and red blood cells [51]. Although primarily expressed in the erythroid lineage rather than immune cells, this transporter is critical for maintaining plasma S1P levels and establishing S1P gradients, and together with other transporters, coordinates both local and systemic S1P distribution [45,51,52,53,54].

### 2.4. S1P Gradient and Immune Cell Trafficking

The S1P gradient plays a crucial role in regulating the egress of immune cells into the blood and lymph and directing their homing to lymphoid organs [55]. An S1P gradient is generated owing to the differences between the level of S1P in the interstitial fluid of tissues (low S1P) and vascular compartments such as blood and lymph (high S1P). Plasma S1P concentrations are high in the micromolar order, while S1P levels in stromal fluid are estimated to be in the nanomolar range [56,57,58,59]. Immune cells detect S1P levels via their cell-surface S1P receptors (S1PRs). Differential expression of these receptors (S1PR1-S1PR5) on immune cell surfaces dictates the attraction of cells to higher concentrations of S1P and can be critical for modulating immune cell differentiation and immune responses to stimuli. For example, S1PR1 is expressed at low levels on the surface of early thymic progenitor cells. These cells cannot respond to the S1P gradient between the thymus and the medullary blood vessels; however, upon negative selection and subsequent S1PR1 upregulation, S1PR1 activation by S1P allows mature T cells to leave the thymus and enter the bloodstream [60,61,62,63,64]. High concentrations of S1P in the blood leads to internalization of S1PR1 on the T cell surface and desensitization to S1P until T cell migrate to lymph nodes containing low levels of S1P maintained by S1P lyase. This allows for subsequent S1RP1 upregulation and sensitization to S1P gradient to exit the lymph node [65]. During pathological events such as inflammation, S1PR1 is regulated post-translationally and transcriptionally during inflammation. Lymphocytes upregulate the early activation marker CD69 in response to type I interferons and other stimuli. CD69 binds surface S1PR1 and induces its internalization, confining naive lymphocytes in inflamed lymph nodes and prolonging their search for cognate antigen [66,67]. After 3 days, T cells can re-upregulate S1PR1 surface expression and resensitize to S1P [68]. Secretion of S1P through Spns2 on lymphatic endothelial cells is then critical for guiding T cells from draining lymph nodes to cutaneous tissues [69]. Although not discussed further in this review, this dynamic relationship between S1P gradient development and S1P receptor expression on immune cells is critical for proper immune cell trafficking.

## 3. S1P Receptors and Immune Cell Signaling

In the late 1990s, a new perspective on the biological role of S1P emerged with the identification of the orphan G protein-coupled receptor, also known as endothelium differentiation gene 1 (EDG1). After the discovery that S1P bound to EDG1, the receptor family was renamed Sphingosine 1-Phosphate Receptor (S1PR) [70,71]. Immune cells exhibit diverse S1PR expression patterns. S1PR1 is constitutively expressed on most immune cells, whereas S1PR2, S1PR3, S1PR4, and S1PR5 have more restricted distributions and expression patterns, leading to differences in S1P responses during activation, maturation, and differentiation.

**Figure 2:**
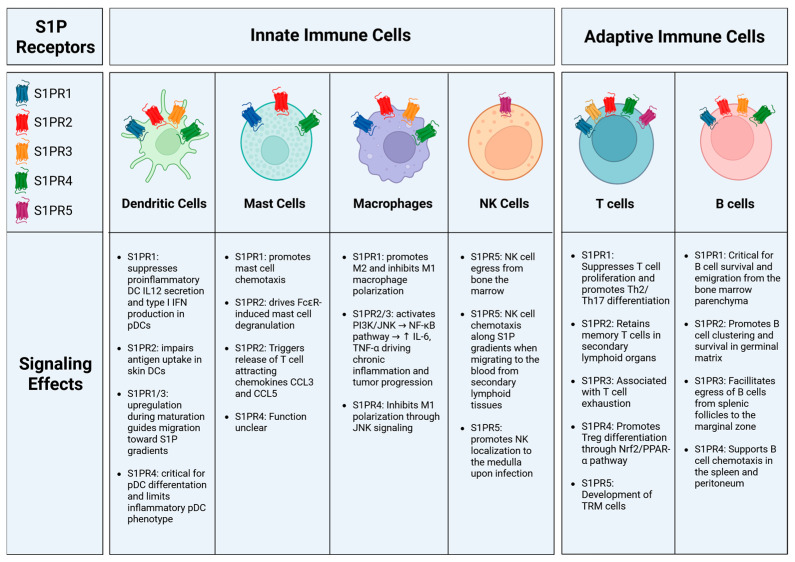
S1P receptor signaling downstream effects on immune cell populations. Immune cell populations exhibit heterogeneity in the S1PRs expressed on their cell surfaces. This leads to different responses to S1P, including cues for cell maturation and migration. Created in BioRender. MEHROTRA, S. (2026) https://BioRender.com/biw9pl0 (accessed on 20 January 2026).

Multiple groups have investigated the signaling mechanisms of S1P receptors in detail. Upon ligand engagement, S1P binding to its G protein-coupled receptors initiates downstream signaling through Gi/o, Gq, or G12/13 pathways. S1PR1 primarily couples to Gi/o proteins, leading to activation of Ras, Akt, Rac, and phosphoinositide 3-kinase (PI3K), while simultaneously suppressing cyclic AMP production. Activation of Ras subsequently drives the extracellular signal-regulated kinase (ERK) signaling cascade [72,73]. S1PR2 primarily binds to G12/13 to activate the low-molecular-weight G protein Rho, and Rho, in turn, activates Rho kinase (ROCK/ROK) [74,75,76]. S1PR3 predominantly conjugates to Gq to activate phospholipase C, trigger calcium mobilization and subsequent protein kinase C activation [77,78]. S1PR4 and S1PR5 both preferentially bind to Gi/o and G12/13 to activate Rho/ROCK and ERK pathways [79,80]. These receptor-specific signaling cascades underpin the diverse functional outcomes of S1P signaling across immune cell types. In many cases, the primary consequence of receptor activation is the regulation of immune cell migration and retention. Notably, low concentrations of S1P promote chemotaxis, whereas higher concentrations exert inhibitory effects. Thus, S1P gradients act as key directional cues that orchestrate immune cell positioning and trafficking. In the sections below, we provide further detail on how these receptors influence immune cell movement and signaling (summarized in Figure 2).

### 3.1. Dendritic Cells

Myeloid cells, such as macrophages and dendritic cells (DCs), typically reside in tissues and enter the lymph circulation to draining lymph nodes upon antigen capture and subsequent activation. The S1PR repertoire expressed before and after activation controls these cells’ responsiveness to S1P. Immature DCs mainly express S1PR2 and S1PR4, while S1PR1 and S1PR3 are strongly stimulated during maturation following antigen uptake, enabling mature DCs to enter the lymphatic system and present the acquired antigens to lymphocytes in the lymph nodes [81]. High concentrations of S1P during DC maturation alter monocyte-derived DC populations via S1PR1 signaling, reducing the secretion of the inflammatory cytokine Interleukin-12 (IL-12) [82,83,84,85]. Furthermore, these altered DCs downregulate the transmembrane protein CD1a and produce high levels of cytokines, including tumor necrosis factor alpha (TNF-α) and IL-10, suggesting that S1P induces the generation of immunosuppressive DCs. Accordingly, loss of S1PR3 signaling in dendritic cells (DCs) impairs their full maturation and skews their immunological function toward a tolerogenic phenotype. S1PR3-deficient DCs exhibit an immature state and preferentially promote Th2 polarization characterized by IL-4 production, rather than driving a Th1/IFN-γ response. Functionally, this shift is associated with the attenuation of inflammatory injury, highlighting that S1P–S1PR3 signaling in DCs is a key regulator of pro-inflammatory Th1 immunity, whereas its absence favors anti-inflammatory Th2 responses in specific pathological contexts, such as ischemia–reperfusion injury [86]. The discovery of FTY720, an S1P receptor functional antagonist that blunts autoimmune disease symptoms, led to subsequent findings that inhibiting S1PR1 signaling reduces the generation of immunogenic DCs and protects against organ injury [87].

Building on this dynamic regulation, S1P receptor expression dynamically regulates dendritic cell (DC) function across different stages of maturation. Signaling through S1PR2 impairs antigen uptake of skin-associated DCs. S1PR2 stimulation modulates the Phosphoinositide 3-kinase (PI3K)/Protein Kinase B (Akt) pathway, reducing Akt phosphorylation and dextran micropinocytosis [88]. This provides a unique platform for DCs to respond to S1P in different states of maturation. Differentiated but immature DCs express high levels of S1PR2 and S1PR4 and self-regulate S1P secretion through ABC transporters. Upon antigen uptake and maturation, S1PR1 and S1PR3 are upregulated, thereby promoting migration toward higher S1P gradients [81]. Mature murine DC migration and endocytosis depend on S1PR3 expression [89]. Consistent with this, Bajwa et al. showed that mice deficient in S1PR3 on bone marrow cells were resistant to ischemic reperfusion injury, and that DCs from these mice lacked maturation and promoted a T-cell regulatory response in a murine model [86,90].

In addition to conventional DCs, Plasmacytoid DCs (pDCs) are a specialized subset of DCs that produce high levels of IFNs and play a key role in the development of autoimmune diseases. Signaling via S1PR1 suppresses these pathogenic DCs by blunting type 1 IFN auto-amplification. Enhanced S1PR1 signaling accelerates IFNAR1 turnover; thus, reducing signal transducer and activator of transcription (STAT) 1 phosphorylation and limiting downstream IFN responses [91]. pDCs dominantly express S1PR4. *S1pr4* deletion in mice does not alter mature pDC migration but reduces common DC progenitor differentiation toward pDC maturation [92]. Signaling through S1PR4 can limit the inflammatory pDC phenotype by preserving the regulatory surface marker Ig-like transcript 7, thereby restricting IFN-α production. Furthermore, reprogramming of pDCs by S1PR4 signaling has downstream effects on T cell differentiation towards regulatory T cells [93]. Signaling through S1PR4 on DCs has also been linked to immunosuppression. In response to tumor secretion of S1P binding to S1PR4, DCs secrete IL-27, which induces a regulatory T cell population generation [94]. Taken together, these findings underscore that distinct S1P receptor axes coordinately regulate DC maturation, migration, and immunoregulatory function, establishing S1P as a key modulator of DC maturation, activation, and migration.

### 3.2. Macrophages

S1P is crucial for macrophage migration and function in physiological and pathological circumstances [34,95,96,97]. Human and murine macrophages express S1PR1-4, although murine macrophages predominantly express S1PR1 and S1PR2 [98,99]. Notably, S1PR expression is dynamically regulated during inflammation, S1PR1 is increased during inflammation resolution to allow macrophage emigration from the site of inflammation, whereas S1PR2 predominates in proinflammatory cells [100,101]. Blocking S1PR1 function using FTY720 alters the macrophage repertoire, inhibiting proinflammatory M1-type macrophages and activating anti-inflammatory M2-type macrophages [102,103,104]. The S1PR2/S1PR3 axis is also critical for polarizing M1-type bone marrow-derived monocytes/macrophages in response to S1P. S1PR2/3 signaling through G protein-coupled receptor alpha leads to PI3K activation, downstream c-Jun N-terminal kinase phosphorylation, and production of TNF-α and chemokine C-C motif chemokine ligand 2 (CCL2) [105]. Meanwhile, S1PR4 restrains M1 macrophage programming by mediating the JNK pathway [106], suggesting alterations in surface S1PR expression can regulate macrophage polarization. Together, these findings indicate that S1P receptor signaling acts as a key regulator of macrophage polarization and functional plasticity. In addition to polarization, S1P impacts many aspects of macrophage phagocytosis, homeostasis, and response to disease [95].

### 3.3. Mast Cells

The function and mechanisms of S1P-S1PR activation in mast cells have been extensively studied using genetic knockout mouse models [34,107,108,109]. S1P is secreted from mast cells following crosslinking of high-affinity IgE Fc receptors (FcεR) and induces transactivation of S1PR1 and S1PR2 [110]. In research employing mast cells generated from embryonic liver progenitors, loss of *SphK1* reduces IgE-induced degranulation and production of chemokine CCL2 and eicosanoid derivatives, suggesting S1P signaling is critical for proper mast cell response [111]. Downstream S1P receptor signaling triggers release of significant amounts of vascular endothelial growth factor A and matrix metalloproteinase-2 [112]. Genetic deletion of *S1pr2* or antisense nucleotide-mediated suppression of *S1pr1* or *S1pr2* reveals that these receptors have distinct, non-overlapping functions in FcεRI-stimulated mast cell degranulation and chemotaxis. Specifically, silencing of *S1pr1* impairs chemotactic motility, while loss of *S1pr2* decreases FcεR-induced mast-cell degranulation of allergic mediators such as histamine [110]. Signaling through S1PR2 is critical for the initiation of acute allergic lung reactions and atopic dermatitis [113,114]. Binding of S1P to S1PR2 leads to STAT3 phosphorylation and release of T-cell-attracting chemokines CCL3 and CCL5. *S1PR2* genetic ablation or S1PR2-specific pharmacological antagonists, such as JTE-013, drastically reduce mast cell activation and secretion of T-cell chemoattractant and mitigate IgE-dependent allergic reactions [115]. Together, these findings underscore the distinct and complementary roles of S1PR1 and S1PR2 in regulating mast cell migration and effector function.

*S1PR4* mRNA has also been detected in mast cells; however, the function of S1PR4 is still controversial. Global loss of *S1pr4* in mice does not impact differentiation of progenitor cells toward mast cell formation or canonical mast cell FcεRI-mediated degranulation in vitro, suggesting this receptor is dispensable [68]. However, CYM50358, a selective agonist of S1PR4, suppresses mast cell degranulation and release of β-hexosaminidase and downstream inflammation in the lungs in an allergic asthma model [116]. These seemingly conflicting findings suggest that S1PR4 may play a context-dependent or modulatory role in mast cell activation. Further studies are required to elucidate the mechanisms underlying S1PR4 targeting in mast cell responses.

### 3.4. NK Cells

The importance of S1P signaling in Natural Killer (NK) cells was first recognized with reduced egress of NK cells from lymph and bone marrow to blood in *S1pr5*-deficient mice or following FTY720 treatment [117,118]. Further studies in Niemann-Pick type C disease highlight the importance of sphingosine storage and S1P gradient on NK trafficking [119]. Together, these findings underscore the central role of S1P gradients in regulating NK cell trafficking. During NK cell differentiation, there is an inverse relationship between C-X-C chemokine receptor type 4 (CXCR4) and S1PR5 expression, with high S1PR5 expression favoring the egress of mature NK cells from the bone marrow. Alternating sensitivity of NK cells to S1P via S1PR5 engagement allows for trafficking of these cells along an S1P gradient from the peripheral immune tissues to the blood [120,121]. Within the lymph node, secretion of S1P by SPNS2 and signaling through S1PR5 is critical for properly localizing NK cells to the medulla and IFNγ production after infection [122]. Thus, S1P–S1PR5 signaling coordinates both NK cell positioning and effector function. However, further studies are needed to elucidate the molecular and cellular mechanisms downstream of S1PR5 that regulate NK cell migration, activation, and functional responses.

### 3.5. T Cells

S1P modulates the biological responses of lymphocytes, most notably T cells. T cells express S1PR1-S1PR5. As discussed above, T cell localization and trafficking are highly correlated with the response to S1P, which is mediated by S1PR1 signaling. Additionally, S1P signaling via S1PR4 has been shown to support trans-lymphatic endothelial migration in T cells [123]. In addition to migration, S1P signaling mediates multiple other T cell functions, including cell survival, proliferation, differentiation, and cytokine production. S1P signaling via S1PR1 maintains mitochondrial content in naive T cells, providing energy to sustain their continuous movement throughout the lymphatic system [124]. T cell receptor activation leads to transcriptional repression of S1PR1 [63]. S1PR1 transcriptional downregulation has also been shown to be essential for the development of Tissue-Resident Memory (TRM) CD8+ T cells [125]. In contrast to S1PR1, S1P signaling through S1PR2 appears to enhance retention of memory T cell subsets in secondary lymphoid organs [126]. S1PR2 co-expression with CD69 maintains CCR6+ gamma-delta T cells in the dermal tissue by limiting S1PR1-dependent egress to the draining lymph node [127]. Similarly, S1PR5 is selectively suppressed in TRM cells and plays a crucial role in T cell infiltration and egress from peripheral organs. Ectopic S1PR5 expression inhibits TRM cell development, whereas deleting S1PR5 improves cutaneous TRM cell development via boosting peripheral T cell retention [128]. Collectively, these findings highlight the central role of S1P receptors in coordinating T cell trafficking and tissue residency.

In addition to regulating cellular localization, S1P suppresses T cell homeostatic proliferation, TCR-induced proliferation, and differentiation. S1PR1-mediated signaling reduces IFNγ production in CD4+ T cells [129,130]. Upregulation of transcription factors c-Maf, Jun-B, and Gata-3 via the S1P-S1PR1 axis also contributes to an enhanced Th2 phenotype [131]. Additionally, overexpression of *S1pr1* on CD4+ T cells promotes the expansion and activity of Th17 cells [132,133]. Impaired phosphorylation and subsequent receptor internalization of S1PR1 directly activate the Janus kinase (JAK)/STAT3 signaling pathway and enhance Th17 polarization. This ultimately exacerbates autoimmune neuroinflammation [134]. Thus, beyond migration, S1P signaling plays a pivotal role in shaping T helper cell differentiation and effector function.

S1P signaling also alters regulatory T cell (Treg) function. Signaling through S1PR1 can selectively activate the Akt/mechanistic target of rapamycin (mTOR) pathway, which functions downstream of IL-2 and limits induced Treg formation while promoting Th1 development. Signaling through SIPR1 further inhibits Smad3 activity, a key signal transducer that controls TGF-β-mediated induced Treg production. This decreases the differentiation of thymic Treg precursors and limits Treg-mediated immunosuppressive activity, disrupting immunological homeostasis [135,136]. In parallel, S1PR1 supports Th17-mediated inflammatory responses, as its deletion in Th17 cells attenuates autoimmune pathology. Conversely, loss or sustained modulation of S1PR1 in Treg cells impairs their stability and survival, collectively shifting the immune balance toward Th1/Th17-driven inflammation [137]. Conversely, signaling through S1PR4 promotes Treg differentiation and enhances fatty acid oxidation through a Nuclear factor erythroid-related factor 2 (Nrf2)/Peroxisome proliferator-activated receptor alpha (PPARα)-dependent pathway, suggesting receptor-dependent responses of T regulatory cells to S1P [138]. Together, these observations indicate that S1P receptor signaling acts as a critical regulator of the balance between effector and regulatory T cell responses. Despite the undeniable importance of S1PRs, much remains to be learned about their role in leukocyte retention in peripheral tissues, how inflammation may alter S1P sensing, and how S1PR signaling is regulated across different T cell populations.

S1P also directly affects several intracellular targets in an S1PR-independent manner. In mouse CD8+ T cells, intracellular S1P generated by SphK1 interacts with peroxisome proliferator-activated receptor gamma (PPARγ) to regulate lipolysis. *Sphk1*-deficient T cells retain their central memory characteristics while increasing mitochondrial respiration and decreasing differentiation to Tregs. In T cell hybridomas, SphK2 interacts with the cytoplasmic region of IL-12 receptor beta 1, and transient SphK2 expression enhances STAT4-mediated transcriptional activation triggered by IL-12 [139]. In vitro, mouse CD4+ T cells lacking *Sphk2* exhibit a hyperactivated phenotype, with markedly increased cytokine secretion and proliferation in response to IL-2 and decreased sensitivity to Treg-mediated inhibition [140]. *Sphk2* knockdown increases Th1-type cytokine production and inflammation in the murine collagen-induced arthritis model [141]. Additionally, CD4+ T cells without *Sphk2* exhibit enhanced activity and proliferation after lymphocytic choriomeningitis virus infection [142]. These findings highlight that S1P signaling extends beyond receptor-mediated effects to include intracellular mechanisms that shape T cell metabolism and function, underscoring the need for further investigation of its intracellular targets to fully realize its therapeutic potential.

### 3.6. B Cells

B cells need S1PR1 and S1PR3 to localize to the appropriate regions in secondary lymphoid organs [143,144,145]. S1PR1 facilitates the emigration of immature B cells from the bone marrow parenchyma into sinusoids, enabling their entry into circulation [146]. Additionally, without S1P signaling, B cell precursors undergo increased apoptosis in the bone marrow [147]. In the spleen, S1P directs B cell movement from follicles to marginal zones through S1PR1-dependent signaling [144,145]. Alternatively, S1PR3 signaling on marginal zone B cells inhibits their flow from the marginal zone toward the follicle, suggesting alternating dynamics of follicular versus marginal B cells in response to S1P [148]. S1P within germinal centers through S1PR2 promotes B cell survival and clustering at the follicular center, aiding in the formation and maintenance of germinal center architecture [149]. S1PR4 is also expressed on B cells, and supports B cell chemotaxis in the spleen and peritoneum [150,151]. Little is known, however, regarding the intracellular signaling that occurs in B cells in response to S1P binding and whether S1P facilitates B-cell function in addition to B-cell migration.

## 4. S1P Alterations of Tumor-Associated Immune Responses

Cancer cells are a primary source of S1P production in the tumor microenvironment (TME). These cells respond to extracellular cues, such as estradiol or extracellular matrix stiffness, to increase S1P production via ERK1/2-mediated activation of SphK1. Tumor cells undergoing apoptosis also upregulate SphK1, thereby increasing S1P production [152]. ABC transporters then secrete S1P, which activates S1P receptors on cells within the tumor niche [39,153]. In addition, other cell types with the TME, such as tumor-associated macrophages and stromal cells, can also secrete S1P [154].

One function of S1P is to act as a chemoattractant “come eat me” signal for antigen-presenting cells; however, within the TME, S1P can additionally modulate immune responses to promote immunosuppression and tumor growth. Increased SphK1 expression in the TME correlates with an increased abundance of suppressive immune cells, including Tregs, tumor-associated macrophages, and myeloid-derived suppressor cells. Increased SphK1 expression also corresponds to increased inhibitory markers on immune cells, such as lymphocyte-activation gene 3 (Lag3), T cell immunoreceptor with Ig and ITIM domains (TIGIT), cytotoxic T-lymphocyte-associated protein 4 (CTLA4), and programmed cell death protein 1 (PD-1) in many different cancer types [155,156]. This ultimately reduces the effectiveness of anti-tumoral immune responses.

### 4.1. Innate Immune Response

The release of S1P by apoptotic cells has significant effects on macrophage polarization, cytokine release, and angiogenesis. S1P released by apoptotic cells also induces the upregulation of anti-apoptotic proteins B-cell lymphoma-extra-large (Bcl-xL) and B-cell lymphoma 2 (Bcl-2) to protect macrophages from cell death [157]. It also stimulates murine macrophages to secrete prostaglandin E2, which induces endothelial cell migration and angiogenesis, key hallmarks of tumor progression [101,152,157,158,159,160]. S1P shifts tumor-associated macrophages (TAMs) toward an M2 phenotype, promoting the release of anti-inflammatory IL-10, and can alter macrophage recruitment to reduce tumor cell killing [161]. High TAM infiltration into tumors has been correlated with poor clinical outcomes in patients and reduced response to chemotherapy and other agents [162,163,164].

A few recent studies have begun to highlight how S1P metabolically polarizes TAMs. S1P can substantially decrease the induction of inducible nitrogen oxide synthase (iNOS) by NF-κB while also increasing ALOX15, thereby inducing an anti-inflammatory phenotype in these macrophages [103,165]. Furthermore, S1P binding to S1PR2 triggers NF-κB and HIFa activation, leading to upregulation of NLRP3 inflammasome. S1P binding also leads to secretion of the inflammatory IL-1β from TAMs, which, in combination with NLRP3 inflammasome activation, suppresses T cell immune anti-tumor responses [162]. TAM phenotype switching towards immunosuppression can be induced via S1P binding to S1PR4 and subsequent activation of peroxisome proliferator-activated receptor, leading to upregulated lipid metabolism [166]. All these studies indicate that S1P may promote tumor progression by inducing macrophage phenotypic switching from the proinflammatory M1 to the anti-inflammatory M2 subtype, thereby facilitating tumor evasion of the host immune system.

In addition to influencing phenotype switching, the S1P/S1PR axis can induce pro-angiogenic properties in murine macrophages, thereby contributing to tumor angiogenesis and, in turn, metastasis. S1PR1 signaling is necessary for the angiotensin II-dependent production of TAMs in the spleen [167]. Direct S1P stimulation also induces immunosuppressive M2 polarization via IL-4 [168], thereby reducing proinflammatory cytokine secretion. S1P binding to macrophages also inhibits iNOS activity and increases arginase I activity [103]. As demonstrated in colitis-associated colon cancer, not only does the phenotype switch from inflammatory M1-like to immunosuppressive M2-like macrophages contribute to cancer progression, but exaggerated macrophage-mediated inflammatory responses may also promote cell transformation and tumor growth.

SphK/S1P/S1PR-dependent activation of macrophages and the resulting proinflammatory cytokine secretion have been identified as significant contributors to chronic intestinal inflammation that leads to cancer development. This has been hypothesized to be partially dependent on macrophages, as SphK1 expression is upregulated following *SphK2* deletion, thereby increasing S1P levels. In this context, intracellular S1P activates NF-κB signaling, leading to the transcription of the proinflammatory genes *il6* and *Tnfa*. Both cytokines induce a vicious cycle that promotes long-lasting inflammation and tumor progression. TNF-α amplifies NF-κB signaling and IL-6 sustains prolonged S1PR1-dependent STAT3 activation [169]. Significantly, S1P-mediated induction of M2 macrophage polarization and angiogenesis correlates with cancer progression [170].

S1P also alters other innate immune cell responses within the tumor. NK cells are highly cytotoxic innate immune cells that secrete TNF-α and IFNγ in the absence of major histocompatibility complex I (MHC-I) expression on tumor cells. However, within the tumor microenvironment, their phenotype switches towards immunosuppressive with increased expression of inhibitory receptor natural killer group (NKG) 2A and reduced expression of S1PR1 [171,172]. Additionally, increased co-expression of S1PR5 and CXCR4 desensitizes these NK cells to cytotoxic stimuli, thus promoting immunosuppression of these cells within the tumor microenvironment [173]. The effects of S1P on other innate immune cells, such as mast cells in the TME, remain to be explored.

### 4.2. Adaptive Immune Response

S1P alters T cell function within the TME. Extracellular vesicles from tumor cells can contain SphK1, leading to extracellular S1P synthesis and upregulation of exhaustion markers on T cells [174,175]. High expression of S1PR3 on T cells in the TME correlates with T cell exhaustion and poor cancer survival outcomes and tumor staging in multiple cancers, while S1PR1 surface expression seems to be critical for anti-tumor responses in the case of glioblastoma but not in medullary adenocarcinoma, suggesting tumor-dependent heterogeneity of S1P-mediated T cell responses [176,177]. S1P–S1PR4 signaling promotes Treg differentiation, which may contribute to immunosuppression within the tumor microenvironment. Higher S1PR4 expression is associated with mammary tumor progression and a concomitant reduction in intertumoral CD8+ T cells [178]. S1PR1 signaling in TILs promotes tumor progression in murine medullary adenocarcinoma by limiting CD8+ T cell recruitment and activation and driving intertumoral Treg accumulation. Tregs, but not CD8+ T cells, are affected by S1PR1 induction. In addition, upregulating S1PR1 in CD4+ T cells induces STAT3 activation and JAK/STAT3-dependent Treg tumor migration, while functionally abrogating STAT3 in T cells reduces tumor-associated Treg accumulation and tumor progression. These murine studies show a striking disparity in the effects of the same signaling receptor, S1PR1, in controlling Tregs in tumors and the periphery [179]. In recent years, a few studies have also suggested that S1PR-independent signaling can alter immunometabolism. S1P generated from SphK1 can lead to PPARγ-mediated suppression of lipolysis in T cells, thereby regulating their differentiation towards regulatory T cell pathways and central memory development [180]. However, the impact of SphK2 and intracellular S1P signaling on T cell function within the TME remains to be explored. Furthermore, although S1PR1 expression is downregulated on B cells in tumor-draining lymph nodes and can alter their migration [181]. Further studies are needed to determine whether S1P influences B cell migration, B cell function, or the formation of tertiary lymphoid structures within the tumor.

Collectively, the studies discussed above illustrate that S1P signaling exerts diverse and sometimes opposing effects on immune cell populations within the tumor microenvironment. S1P can regulate multiple aspects of tumor immunity, including immune cell trafficking, differentiation, and functional activation across both innate and adaptive immune compartments. However, the biological outcomes of S1P signaling often appear context-dependent, with studies reporting both pro-tumor and anti-tumor immune effects. These seemingly contradictory findings likely reflect the complexity of the S1P signaling axis, which integrates multiple regulatory variables, including the cellular sources of S1P, the repertoire of S1P receptors expressed by immune cells, and the metabolic and inflammatory conditions within the TME. To better synthesize these diverse observations, it is useful to consider S1P signaling within a broader mechanistic framework that links S1P production, receptor-mediated signaling pathways, and downstream immune cell responses.

### 4.3. Therapeutic Approaches to Target S1P in the Tumor Microenvironment

**Figure 3:**
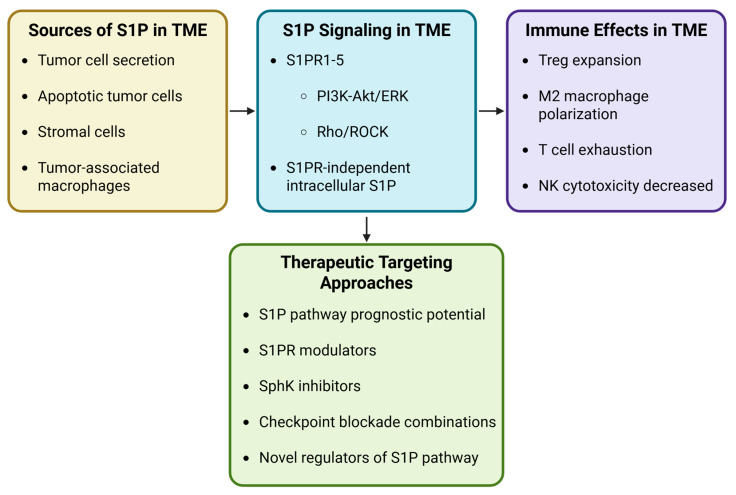
Mechanistic Framework for S1P-Mediated Immune Regulation in Cancer. Created in BioRender. MEHROTRA, S. (2026) https://BioRender.com/7totfd9 (accessed on 20 January 2026).

S1P signaling within the TME is a dynamic regulatory network that integrates lipid metabolism, receptor signaling, and immune cell function. Rather than acting as a uniformly pro- or anti-tumor factor, the immunological outcome of S1P signaling is determined by several interacting variables, including the cellular source of S1P, the repertoire of S1P receptors expressed by immune cells, and the metabolic state of the TME (Figure 3).

Tumor cells, stromal cells, and tumor-associated macrophages can contribute to S1P production through sphingosine kinase activity, establishing gradients that regulate immune cell trafficking and positioning within tumors. Activation of S1P receptors (S1PR1–5) triggers downstream signaling pathways including PI3K–Akt, ERK and Rho/ROCK that influence immune cell migration, differentiation, and survival. In addition to receptor-dependent signaling, intracellular S1P has been shown to regulate metabolic and transcriptional programs that shape immune cell fate. Together, these mechanisms form a context-dependent regulatory axis that can promote either anti-tumor immunity or immune suppression within the TME.

Building on the mechanistic insights described above, the SphK/S1P signaling axis has emerged as an attractive therapeutic target for modulating immune responses within the tumor microenvironment. Pharmacological strategies aimed at inhibiting S1P production through SphK inhibitors or modulating S1PR signaling are therefore being actively explored. These approaches seek to reshape tumor-associated immune responses and enhance anti-tumor immunity.

Tumor-infiltrating immune cells, including DCs, T cells, neutrophils, NK cells, and macrophages, are critical for cancer treatment efficacy and prognosis [182]. The S1P gradient is essential for lymphocyte egress to the tumor site. Therefore, modulation of S1P is a promising target for cancer therapy. However, caution and further in-depth analysis of the tumor microenvironment are warranted before prescribing S1P modulators. In recent years, there has been a deeper understanding of the heterogeneity of S1P markers across cancer types, which can provide additional insight into which patient subsets may respond to S1P modulator therapy [156,183,184,185]. For example, S1PR1 shows heterogeneous expression across bladder, lung, renal cell carcinoma, and head and neck cancers, with expression associated with opposing responses and predicted overall survival in patients. In head and neck cancers, lower *S1PR1* expression correlated with poor survival, whereas in bladder cancer, high *S1PR1* expression correlated with poor survival [183]. Additional S1P pathway markers, including S1PR3, lipid phosphate phosphatase 3 (LPP3), and S1P phosphatase (SGPP1), in combination with S1PR1, may gain additional insight into predicting therapeutic response, overall patient survival, and tumor metastasis [182,186,187,188]. In a retrospective study of patients with triple-negative breast cancer, high expression of *SGPP1* and *LPP3* was associated with increased infiltration of CD4+ and CD8+ T cells, macrophages, neutrophils, and dendritic cells into the tumor. In contrast, low levels of *SGPP1* and *PLPP3* have been associated with poor cancer prognosis [182,189]. In colorectal cancer patients, regulation of S1P signaling through *ACTL6B* expression correlated with increased response to therapy. *ACTL6B* directly dampens S1P signaling by downregulating S1PR3, which, in turn, is associated with increased cytotoxic CD4+ T cell infiltration [190]. Therefore, using S1P signaling as a prognostic marker for therapeutic response to S1P modulators, chemotherapy, and other immunotherapies warrants further investigation.

S1P’s role as a modulator of immune responses to cancer is perplexing and may be highly dependent on S1P levels within the TME and S1PR expression on tumor and immune subsets. Additionally, although we only discuss S1P in the context of immune cells, S1P signaling affects many cellular processes, including proliferation, survival, and differentiation, across diverse cell types in the body [191]. Thus, globally targeting this pathway can have many off-target effects. For example, although FTY720, a global S1P functional antagonist, has been approved for autoimmune diseases and has shown promise in in vitro and murine cancer studies, its off-target effects, including lymphopenia and bradycardia, preclude it as a viable therapeutic strategy [192]. Additionally, some research articles suggest that globally disrupting S1P signaling limits immune capacity to target the tumor. In the case of gastric tumor, FTY720 promotes tumor growth by enhancing the proliferation of MDSCs and granulocyte-macrophage colony-stimulating factor secretion, which are known to disrupt cytotoxic T-cell function [193]. Another disadvantage of globally targeting S1PRs is their heterogeneous expression across multiple tissue types. Thus, utilizing S1P strategies to suppress tumor growth should not be a one-drug-fits-all approach but rather be fine-tuned to the tumor subtype and tumor microenvironment.

Targeting individual S1PRs may be a more viable approach to improving tumor control. S1PR1 selective modulators have been developed with varying responses. SEW-2871, a S1PR1-selective agonist, has differential effects in tumors. It promotes gastric tumor growth by recruiting immunosuppressive MDSCs; however, it has also been shown to enhance NK-mediated tumor lysis [194,195]. Another S1PR1 selective agonist found in ginseng, Ginsenoside F4, enhances DC-mediated antitumor T cell responses, reducing colorectal cancer cell growth in an in vivo mouse model [196]. W146, an S1PR1-specific antagonist, has been shown to improve tumor control by enhancing natural killer T cell-mediated lysis of tumor cells [197]. These differing responses to S1P modulators may be due to differences in S1PR levels on immune cells within the TME. For example, increased S1PR4 expression on DCs was associated with increased immune cell activation and infiltration [189,198]. However, S1PR4 ablation reduces tumor growth and improves chemotherapy via CD8+ T cell expansion [178]. Furthermore, treatment of T cells with CYM50308, an S1PR4 agonist, increased CXCR4 expression, a potent regulator of T-cell migration, on CD8+ low T cells in the TME [189]. This suggests that S1PR4 signaling has dual, opposing modulatory roles depending on the immune cell subset. In breast and colon cancer models, combining epithelial cell adhesion molecule-targeted CAR-T cells with S1PR3 inhibition enhances anti-tumor efficacy by reducing T cell exhaustion and recruiting proinflammatory macrophages to remodel the TME [176]. These approaches target receptor modulation and downstream signaling; however, S1P can also act intracellularly in immune cells [180]. Therefore, by only blocking S1PR-dependent signaling, critical S1P-immune-mediated pathways that are not considered may lead to variability in response.

As our understanding of the regulation of the S1P synthesis pathway increases, intratumoral approaches blocking S1P synthesis may be a viable alternative. One approach is targeting SphK1/2, to modulate S1P production and improve tumor control. SphK1 overexpression in Diffuse large B-cell lymphoma promotes chemoresistance and immune escape while its inhibition enhances doxorubicin efficacy and mediates CD8+ T cell antitumor immunity [199]. SphK1-generated S1P impairs T cell metabolic fitness and promotes Treg differentiation via PPARγ. Inhibition of SphK1 with PF543 enhances T cell central memory phenotype and antitumor activity in melanoma models, supporting SphK1 as a therapeutic target in adoptive T cell therapy [180]. SphK1 also drives immune evasion in melanoma by transcriptionally regulating Programmed Death-Ligand 1 (PD-L1) via the transcriptional repressor metastasis-associated 1 family member 3, leading to reduced immunotherapy response. Inhibiting SphK1 significantly improves anti-PD-1 efficacy in multiple murine models, underscoring its potential as both a therapeutic target and a predictive biomarker for immune checkpoint blockade [155,200]. SphK1 can be secreted via extracellular vesicles, leading to T cell exhaustion and upregulation of PD-L1 on tumor cells. Combining SphK1 inhibition with PF543 and anti-PD-1 therapy synergistically reduced tumor burden and metastasis in ovarian cancer models, suggesting that combining S1P modulators with immunotherapy or chemotherapy may provide greater benefit to patients [175].

Complementary to these findings, inhibiting related enzyme SphK2 with Opaganib induces immunogenic cell death, elicits cross-tumor immunity, and synergizes with checkpoint inhibitors to enhance antitumor efficacy and survival in murine models [201]. Additionally, Opaganib, upon completion of two phase II clinical trials for cholangiocarcinoma (NCT03377179) and castration-resistant prostate cancer (NCT04207255), received orphan drug designation from the FDA for cholangiocarcinoma and neuroblastoma. Therefore, targeting SphKs may be a safer alternative to S1PR-targeting strategies.

In addition to directly targeting sphingosine kinases, targeting upstream regulators of the S1P synthesis pathway or S1PR expression has also shown promise. Researchers found that tumor-associated macrophages expressing high levels of NIMA-related kinase 2 (NEK2) correlated with more advanced stage Hepatocellular carcinoma and worse progression-free survival in patients. NEK2 is essential for TAM immunosuppressive function and activates serine palmitoyl-coenzyme A transferase, a rate-limiting enzyme in S1P biosynthesis. Inhibiting NEK2 in a humanized mouse model reduced S1P secretion by TAMs and could reprogram the TME, thereby improving tumor control in combination with immune checkpoint blockade [154].

In another study, researchers found that S1PR1 was downregulated on the surface of T cells, leading to poor infiltration in murine glioblastoma models. T cell S1PR1 externalization could be rescued by overexpressing the *ATP11B* plasmid in these cells, thereby reprogramming T cells toward an anti-tumor phenotype. Furthermore, these *ATP11B* overexpression plasmids could be specifically targeted to T cells using a cationic liposome nanoparticle conjugated to an anti-CD3 antibody delivery system [177]. Novel strategies to fine-tune S1P-targeted modulators in combination with other immunotherapy approaches may be integral to improving tumor therapy for patients. Lung cancer patients who do not respond to PD-1/PD-L1 therapy have down-regulated secretion of S1P. Researchers subsequently developed an S1P-containing sodium alginate hydrogel for intratumoral injection that releases S1P and anti-PD-L1. In a lung cancer murine model, this approach increased M1-like polarized TAMs within the TME and inhibited tumor growth [202]. Identifying further regulators of S1P synthesis and S1PR-independent mechanisms may lead to novel therapies to further improve cancer patient outcomes.

### 4.4. Outstanding Questions and Future Directions in S1P Immunology

Despite advances in understanding S1P signaling in immune regulation and cancer biology, several key questions remain unresolved. What determines the dual pro- and anti-tumor roles of S1P signaling in different tumor contexts? We have published earlier that S1PR-independent intracellular S1P signaling that alters T cell immunometabolism; however, how intracellular S1P regulates immune cell metabolism and transcriptional programs across different immune subsets remains incompletely understood. Furthermore, S1P gradients are generated in the tumor; however, how these gradients are spatially organized within the tumor and how this influences immune cell positioning and/or exclusion remains unknown. There are a few S1PR-specific targeting strategies that have shown promise in murine models; however, whether these strategies translate into improved tumor control and patient outcomes in the clinic still needs to be evaluated. Furthermore, developing more specific, S1PR-independent therapeutics should be explored to reduce the global off-target effects of S1P modulators. Finally, how modulation of the SphK/S1P axis can be integrated with emerging immunotherapies, such as immune checkpoint blockade or adoptive cell therapy in clinical trials, remains to be determined. Addressing these questions will help clarify the complex immunological roles of S1P signaling and guide the development of next-generation therapeutic strategies targeting the S1P axis in cancer.

## 5. Conclusions

The S1P signaling pathway is an intriguing and intricate target in cancer biology and immune regulation. It orchestrates a wide range of immune functions through its five G protein-coupled receptors, influencing immune cell trafficking, activation, and polarization within the TME, thereby contributing to immune evasion and tumor progression. Therapeutic strategies aimed at modulating S1P signaling—whether through selective S1P receptor antagonists, sphingosine kinase 1/2 inhibitors, or antibody-based neutralization of extracellular S1P—have demonstrated efficacy in preclinical models, with some advancing to early-phase clinical trials. Notably, agents such as PF543 and Opaganib, SphK1 and SphK2 inhibitors, respectively, have shown promise in restoring T cell function, inducing immunogenic cell death, and enhancing the efficacy of checkpoint blockade therapies. However, the pleiotropic effects of S1P signaling and the limited receptor selectivity of currently available modulators, such as FTY720, pose significant challenges related to off-target immune suppression and toxicity. Future research must focus on developing highly selective S1PR modulators. Structural insights, such as the S1P5-inverse agonist complex, enable precision drug design with improved safety profiles.

Beyond its receptor-mediated effects, intracellular S1P influences the fate, metabolism, and function of immune cells through poorly defined pathways. The specific intracellular targets of S1P in immune cells remain largely undefined, limiting our ability to precisely exploit this axis. Furthermore, the dual role of S1P as both an immune activator and an immunosuppressive signal is perplexing and underlines the need for context and tumor-specific therapeutic approaches. Overcoming current knowledge gaps will be crucial to refining our understanding of S1P biology, fully realizing its potential when combined with existing cancer immunotherapies and aiding in the rational design of next-generation immunotherapies that harness or modulate lipid signaling for durable cancer control.

## Data Availability

No new data was generated or analyzed in this review.

## Abbreviations

The following abbreviations are used in this manuscript:

ABC	ATP-binding cassette
Akt	Protein kinase B
AMP	Adenosine monophosphate
Bcl-2	B-cell lymphoma 2
Bcl-xL	B-cell lymphoma-extra large
CAR-T	Chimeric antigen receptor T cell
CCL	C-C motif chemokine ligand
CCR6	C-C chemokine receptor type 6
CD	Cluster of differentiation
CD69	Early T-cell activation marker
CTLA4	Cytotoxic T-lymphocyte-associated protein 4
CXCR4	C-X-C chemokine receptor type 4
DC	Dendritic cell
EAE	Experimental autoimmune encephalomyelitis
EGFP	Enhanced green fluorescent protein
ERK	Extracellular signal-regulated kinase
eDG1	Endothelium differentiation gene 1
FcεR	High-affinity IgE receptor
FTY720	Fingolimod
GATA3	GATA-binding protein 3
IFN-a	Interferon alpha
IFN-y	Interferon gamma
IFNAR1	Interferon alpha/beta receptor 1
IL	Interleukin
iNOS	Inducible nitric oxide synthase
iTreg	Induced regulatory T cell
JAK	Janus kinase
Lag3	Lymphocyte activation gene 3
LCMV	Lymphocytic choriomeningitis virus
MAPK	Mitogen-activated protein kinase
MCP-1	Monocyte chemoattractant protein 1
MDSC	Myeloid-derived suppressor cell
MFS	Major Facilitator Superfamily
Mfsd2b	Major facilitator superfamily domain-containing protein 2b
MHC-I	Major histocompatibility complex class I
MRP1	Multidrug resistance protein 1
mTOR	Mechanistic target of rapamycin
NF-kB	Nuclear factor kappa B
NK	Natural killer cell
NKG2A	Natural killer group 2A receptor
Nrf2	Nuclear factor erythroid-related factor 2
pDC	Plasmacytoid dendritic cell
PD-1	Programmed cell death protein 1
PD-L1	Programmed death-ligand 1
PI3K	Phosphoinositide 3-kinase
PLPP	Lipid phosphate phosphatase
PPARα	Peroxisome proliferator-activated receptor alpha
PPARγ	Peroxisome proliferator-activated receptor gamma
Rac	Ras-related C3 botulinum toxin substrate
Ras	Rat sarcoma small GTPase
Rho	Ras homolog small GTPase
ROCK	Rho-associated kinase
S1P	Sphingosine 1-phosphate
S1PR	Sphingosine 1-phosphate receptor
SGPP	Sphingosine 1-phosphate phosphatase
SphK	Sphingosine kinase
Spns2	Spinster homolog 2
STAT	Signal transducer and activator of transcription
TAM	Tumor-associated macrophage
Tbet	T-box transcription factor TBX21
TCR	T-cell receptor
TGF-B	Transforming growth factor beta
Th	T helper cell
Tigit	T cell immunoreceptor with Ig and ITIM domains
TME	Tumor microenvironment
TNF-a	Tumor necrosis factor alpha
TRM	Tissue-resident memory T cell
Treg	Regulatory T cell
